# Clinical Characteristics of Predominantly Unilateral Oral Cenesthopathy With and Without Neurovascular Contact

**DOI:** 10.3389/fneur.2021.744561

**Published:** 2021-09-20

**Authors:** Kazuya Watanabe, Motoko Watanabe, Chihiro Takao, Chaoli Hong, Zhenyan Liu, Takayuki Suga, Trang Thi Huyen Tu, Junichiro Sakamoto, Yojiro Umezaki, Tatsuya Yoshikawa, Miho Takenoshita, Akihito Uezato, Haruhiko Motomura, Tohru Kurabayashi, Yoshihiro Abiko, Akira Toyofuku

**Affiliations:** ^1^Department of Psychosomatic Dentistry, Graduate School of Medical and Dental Sciences, Tokyo Medical and Dental University, Tokyo, Japan; ^2^Department of Physical Therapy, Shimonoseki Nursing and Rehabilitation School, Yamaguchi, Japan; ^3^Department of Basic Dental Sciences, Faculty of Odonto-Stomatology, University of Medicine and Pharmacy, Ho Chi Minh City, Vietnam; ^4^Department of Oral and Maxillofacial Radiology, Graduate School of Medical and Dental Sciences, Tokyo Medical and Dental University, Tokyo, Japan; ^5^Section of Geriatric Dentistry, Department of General Dentistry, Fukuoka Dental College, Fukuoka, Japan; ^6^School of Health and Welfare, International University of Health and Welfare, Tochigi, Japan; ^7^Division of Oral Medicine and Pathology, School of Dentistry, Health Sciences University of Hokkaido, Hokkaido, Japan

**Keywords:** oral cenesthopathy, delusional disorder somatic type, neurovascular contact, trigeminal nerves, Oral DRS, magnetic resonance imaging, burning mouth syndrome, trigeminal neuralgia

## Abstract

Oral cenesthopathy (OC) is characterized by unusual oral discomfort without corresponding evidence, and it has often been categorized as “delusional disorder, somatic type”. Regarding possible causative factors of OC, involvement of neurovascular contact (NVC) of the trigeminal nerve, which transmits not only pain but also thermal, tactile, and pressure sensations, has never been observed yet. This study aimed to investigate the relationship between clinical characteristics of unilateral OC and the presence of trigeminal nerve NVC. This is a retrospective comparative study that involved 48 patients having predominantly unilateral OC who visited the Psychosomatic Dentistry Clinic of Tokyo Medical and Dental University between April 2016 and February 2019. Magnetic resonance imaging was performed to assess NVC presence. The Oral Dysesthesia Rating Scale (Oral DRS) was used to assess the various oral sensations and functional impairments besides psychometric questionnaires. Clinical characteristics were retrospectively obtained from the patients' medical charts. NVC was present in 45.8% (22/48) of the patients. There was no significant difference in sex, age, psychiatric history, oral psychosomatic comorbidity, and psychometric questionnaire scores between patients with and without NVC. However, compared to the patients with NVC, the patients without NVC had significantly higher scores for overall subjective severity of OC symptoms (*p* = 0.008). Moreover, patients having predominantly unilateral OC without NVC showed significantly higher scores in symptom severity and functional impairment of the following parameters: movement (*p* = 0.030), work (*p* = 0.004), and social activities (*p* = 0.010). In addition, compared with the patients with NVC, the patients without NVC showed significantly higher averages of the total symptom severity scale (SSS) and functional impairment scale (FIS) scores in the Oral DRS (*p* = 0.015 and *p* = 0.031, respectively). Furthermore, compared with the patients with NVC, the patients without NVC had significantly higher numbers of corresponding symptoms in both the SSS and FIS (*p* = 0.041 and *p* = 0.007, respectively). While NVC may be involved in the indescribable subtle OC symptoms, more complex mechanisms may also exist in OC patients without NVC, which yield varying and more unbearable oral symptoms.

## Introduction

Oral cenesthopathy (OC) is characterized by unusual discomforts in the oral cavity without corresponding medical or dental evidence. Patients having OC present with various oral sensations, including a slimy/sticky sialorrhea, xerostomia, dysgeusia, and roughness/granularity sensations (1, 2). On the one hand, the OC symptoms overlap with pain sensations similar to burning mouth syndrome (BMS) (3). On the other hand, some OC patients complain of bizarre, strange but real sensations such as the existence of coils, wires, and bubbles, which can be even accompanied by movement and that show similarity to “delusional disorder, somatic type (DDST)” (4), “delusional disorder, unspecified,” or “schizophrenia, episode unspecified” (5). For the overlapping gray zone of such spectrum symptoms, the diagnoses are sometimes difficult to distinguish; however, OC has not entirely satisfied any of them. Previously, we constructed the Oral Dysesthesia Rating Scale (Oral DRS) to assess the various oral sensations that might overlap (2, 6). Moreover, the severity of oral symptoms also varies. Some patients extensively experience oral symptoms in their daily lives, which reduce their quality of life. To assess such varying severities, the scales for the functional impairments are also contained in Oral DRS.

The involvement of the central nervous system (CNS) and the peripheral nervous system has been recently considered to underlie the pathophysiology of abnormal orofacial sensations. Regarding the pathophysiology of OC, a study using single-photon emission computed tomography (SPECT) reported right-dominant asymmetry of the cerebral blood flow (CBF) in the callosomarginal, precentral, and temporal regions in patients having OC (7). Moreover, significant right-dominant CBF asymmetry in the temporal and posterior cerebral regions has been reported regardless of the depression history (8). Therefore, right-dominant asymmetric CBF could be among the specific mechanisms underlying the OC symptoms.

Furthermore, since dental treatments often trigger OC symptoms (2), some stimulus in the peripheral nervous system might be involved. The trigeminal nerve is the largest cranial nerve and is considered to be crucially involved in the transmission of peripheral stimuli to the CNS. This nerve has a sensory and motor function in the facial area, including the oral cavity. The root entry zone (REZ) is where trigeminal nerves enter or exit the brain stem *via* the cisternal portion at the height of the pons. Arterial or venous contact of the trigeminal nerve can occur at the REZ. Neurovascular contact (NVC) of the trigeminal nerve sometimes causes abnormal pain sensations, including trigeminal neuralgia (9) and persistent idiopathic facial pain (PIFP) (10). Trigeminal nerves transmit not only pain but also thermal, tactile, and pressure sensations; therefore, NVC of trigeminal nerves may be involved in other abnormal oral sensations besides abnormal pain. We hypothesized that NVC presence is involved in oral symptom variations, including comorbid pain sensations in patients having OC. Trigeminal nerves mainly decussate and run to the contralateral thalamic nuclei, which project to the cortical region through the thalamocortical tract (11). Given this anatomical nerve tract, we further hypothesized an association between OC symptoms and NVC laterality.

This study aimed to investigate the relationship between the clinical characteristics of patients having predominantly unilateral OC and the presence of trigeminal nerve NVC based on magnetic resonance imaging (MRI) findings.

## Methods

### Patients

This was a retrospective comparative study that involved patients having predominantly unilateral OC who visited the Psychosomatic Dentistry Clinic of Tokyo Medical and Dental University between April 2016 and February 2019.

**Figure 1:**
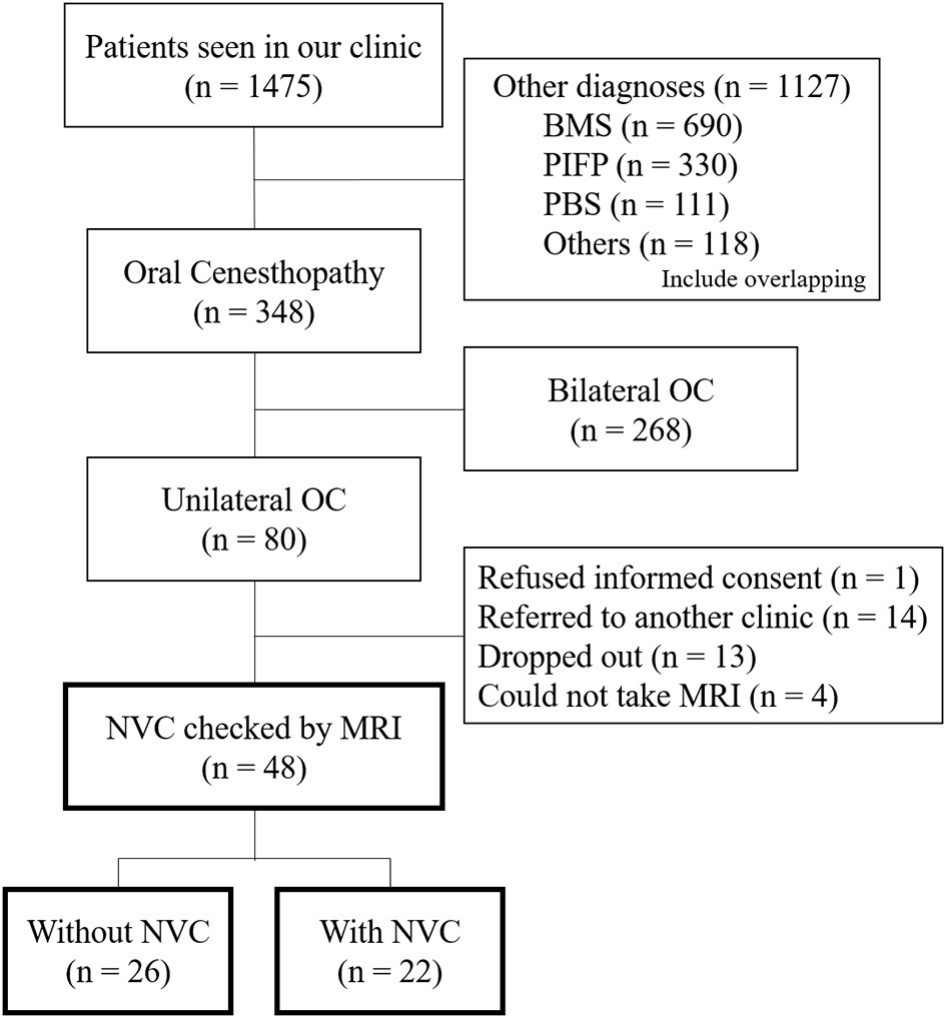
A flowchart of the patients' selection. Flowchart showing the selection of the patients having predominantly unilateral oral cenesthopathy for inclusion in the present study. OC, oral cenesthopathy; MRI, magnetic resonance imaging; NVC, neurovascular contact; BMS, burning mouth syndrome; PIFP, persistent idiopathic facial pain; PBS, phantom bite syndrome.

From the patients' first visit to our clinic (*n* = 1,475), the patients having BMS (*n* = 690), PIFP including atypical odontalgia (*n* = 330), phantom bite syndrome (*n* = 111), or other diagnoses (*n* = 118) were excluded (Figure 1). The diagnoses were performed by specialists in psychosomatic dentistry who were certified by the Japanese Society of Psychosomatic dentistry. For the patients who showed overlapping OC and other diagnoses, their symptoms they were most suffering from were prioritized for the diagnoses and the other overlapping sensations were diagnosed as the comorbid diagnosis. The laterality of OC symptoms was assessed depending on the predominance of symptom appearance. The inclusion criteria were as follows: (1) predominantly unilateral OC symptoms; (2) not better accounted for by other systemic etiology or oral diseases. The exclusion criteria were patients who (1) did not provide informed consent, (2) were referred to another clinic, (3) dropped out, and (4) could not undergo MRI due to the contraindication for MRI.

Clinical characteristics were obtained from the patients' medical charts, including demographic information (sex and age), illness duration, psychiatric history, comorbid oral psychosomatic symptoms, and symptom triggers. All examiners in this study were well-experienced and trained clinicians and researchers.

All the patients provided written informed consent. This study was approved by the Ethical Committee of Tokyo Medical and Dental University Dental Hospital (approval number: D2013-005).

### MRI Protocol and Definitions

All the patients underwent MRI within 1 month after the initial medical examination. All MR images were obtained with 3-Tesla MRI scanner (Magnetom Spectra, Siemens Healthcare, Erlangen, Germany) and a 16-channel head coil. MR sequences were acquired at the REZ level of trigeminal nerves using the same parameters as those in our previous study (10). MR angiography (MRA) was acquired using 3D time-of-flight (3D-TOF) MRA with the following parameters: repetition time (TR)/echo time (TE), 24/3.9 ms; flip angle, 18°; field of view (FOV), 160 mm × 160 mm; matrix, 320 × 192; section thickness, 0.5 mm; and slab number, 3. This was reconstructed to a voxel size of 0.5 mm × 0.5 mm × 0.5 mm and a slab thickness of 44 mm. MR cisternography was obtained through 3D constructive interference in steady-state (3D-CISS) using the following parameters: TR/TE, 7.4/3.7 ms; flip angle, 50°; FOV, 160 mm × 160 mm; matrix, 320 × 320; section thickness, 0.5 mm. This was reconstructed to a voxel size of 0.5 mm × 0.5 mm × 0.5 mm and a slab thickness of 44 mm.

All 3D-TOF and 3D-CISS images were presented in triplanar views (transverse, coronal, and sagittal views) on the visualization system, and experienced oral radiologists blinded to the symptom side evaluated NVC presence. NVC presence was defined as contact between a blood vessel and trigeminal nerves at the REZ without visible cerebrospinal fluid between them in the triplanar 3D-CISS images. The responsible blood vessel type (artery or vein) was evaluated using the triplanar views and memory-in-pixel display of 3D-TOF MRA. Disagreements or uncertainties regarding whether there was contact were indicated as “no NVC” in the data analysis.

### Assessment of Oral Cenesthopathy and Psychological State

OC symptoms were assessed using the Oral DRS, which was developed based on the literature review and extensive clinical experience, to reorganize and objectify the complex symptoms (6). The Oral DRS was composed of the symptom severity scale (SSS), functional impairment scale (FIS), and visual analog scale (VAS). In the SSS [A], oral symptoms were categorized as follows: feelings of foreign body [A1], exudation [A2], squeezing–pulling [A3], movement [A4], misalignment [A5], pain [A6], and spontaneous thermal sensation or tastes [A7]. The FIS [B] assesses the impairment degree of eating [B1], articulation [B2], work [B3], and social activities [B4]. The VAS [C] is composed of two scales that assess overall subjective symptom severity [C1] and changes in symptom severity [C2]. The total score of the SSS and FIS was calculated as the sum of the scores in [A1] to [A7] and [B1] to [B4], respectively. Furthermore, we counted the number of corresponding categories for the SSS and FIS, respectively. The Oral DRS is available in Japanese and English with the respective instruction manuals on the website: http://www.tmd.ac.jp/grad/ompm/details8.html.

The mental and psychological conditions were assessed using two questionnaires. The Zung Self-Rating Depression Scale (SDS) was used to assess the depression status (12, 13), while the Short Intolerance Of Uncertainty Scale (SIUS) was used to assess the uncertainty intolerance tendency (14, 15). The Somatic Symptom Scale 8 (SSS-8) was used to assess the comorbidities of functional somatic symptoms. The eight descriptors that comprise the SSS-8 were rated to evaluate the mental and psychological effects on functional somatic symptoms (16, 17).

Pain catastrophizing was assessed using the Pain Catastrophizing Scale (PCS) (18, 19). Moreover, disability caused by chronic sensation was assessed using subgrouping for targeted treatment generic (STarT-G) (20). Furthermore, illness severity was objectively assessed using the Clinical Global Impression (CGI)-Severity scale (21).

### Analysis

All data analyses were performed using Student's *t*-test, Mann–Whitney U test, and Fisher's exact test. Between-group comparisons of the clinical characteristics were performed using Student's *t*-test (age and illness duration), Mann–Whitney U test (CGI), and Fisher's exact test (the other demographic and clinical characteristics). Regarding questionnaire analysis, Student's *t*-test was used for between-group comparisons of the scores of SDS, SSS-8, SIUS, PCS, and STarT-G. Furthermore, between-group comparisons of the Oral DRS scores and the total number of corresponding parameters were performed using the Mann–Whitney U test, except that VAS and the total scores of SSS and FIS used the Student's *t*-test. The data represent the mean ± standard deviation (SD) or median [interquartile range (IQR)]. All analyses were performed using IBM SPSS Statistics version 25.0 (IBM Corporation, Armonk, NY, USA). Statistical significance was set at *p* < 0.05.

## Results

### Study Participants

From April 2016 to February 2019, a total of 1,475 patients visited the Psychosomatic Dentistry Clinic of Tokyo Medical and Dental University Hospital. Among them, 348 patients were diagnosed with OC, with 80 patients having predominantly unilateral OC. After the exclusion of 32 patients according to the exclusion criteria, 48 patients underwent MRI and assessment of NVC presence (Figure 1).

### Demographic and Clinical Characteristics

**Figure 2:**
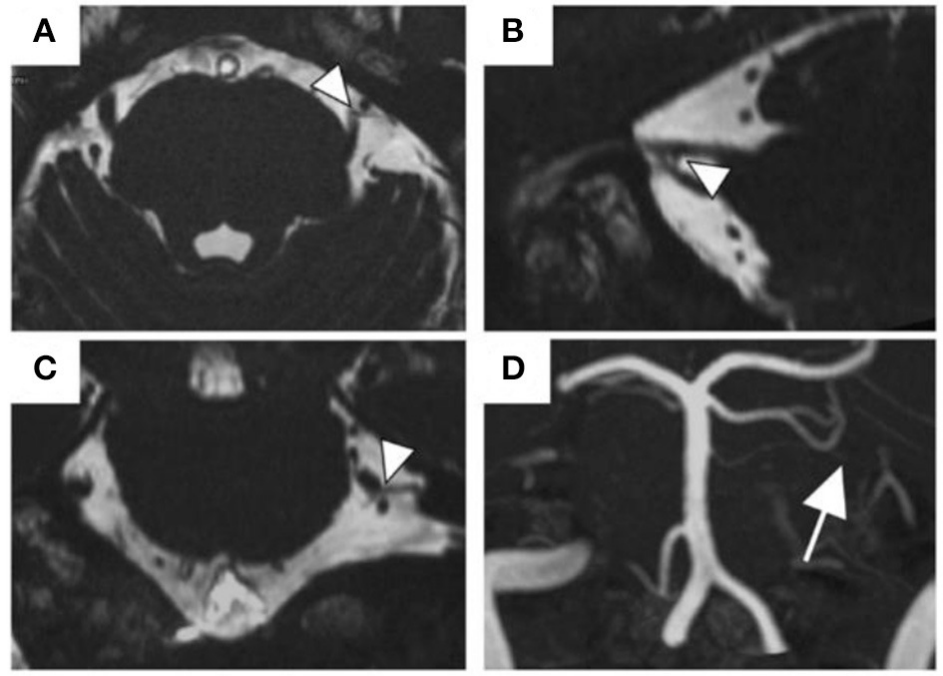
The MR images of NVC of the trigeminal nerves. A 57-year old man with NVC of the trigeminal nerves (arrowheads). The superior cerebellar artery was the responsible blood vessel (arrow). **(A)** Transverse view of the 3D-CISS image, **(B)** sagittal view, **(C)** coronal view, **(D)** MIP display of 3D-TOF MRA. Abbreviations: MR, magnetic resonance; NVC, neurovascular contact; 3D-CISS, 3D constructive interference in steady-stat; MIP, maximum intensity projection; 3D-TOF MRA, 3D time-of-flight magnetic resonance angiography.

NVC of trigeminal nerves was observed in 22 (45.8%) patients having predominantly unilateral OC (Table 1). Figure 2 presents one patient who presented NVC of the trigeminal nerves. Both groups had a high female prevalence with no significant between-group difference [80.8% (21/26) vs. 77.3% (17/22)]. Regarding the predominant symptom side, 72.9% (35/48) of the patients presented symptoms on their left with no significant between-group difference (*p* = 1.000). Among the patients with NVC, 68.2% were ipsilateral to the predominant symptom side and 13.6% were contralateral, while 18.2% showed bilateral NVC. There was no significant between-group difference in sex, age, illness duration, CGI severity, presence of psychiatric history, presence of comorbid oral psychosomatic symptoms, and presence of symptom triggers.

**Table 1:** Demographic and clinical characteristics of the patients having predominantly unilateral oral cenesthopathy.

	**Total**	**Without NVC**	**With NVC**	***p*-value**
	**(*n* = 48)**	**(n = 26, 54.2%)**	**(n = 22, 45.8%)**	
Female (%)^†^	38 (79.2)	21 (80.8)	17 (77.3)	1.000
Age (mean ± SD)^§^	63.2 ± 12.3	63.8 ± 13.6	63.4 ± 12.0	0.915
Duration of illness (mean ± SD, months)^§^	47.1 ± 51.2	56.3 ± 59.6	39.1 ± 43.1	0.254
CGI (median)^‡^	4	4	4	0.501
Predominant symptom side^†^				1.000
Left (%)	35 (72.9)	19 (73.1)	16 (64.0)	
Right (%)	13 (27.1)	7 (26.9)	6 (36.0)	
NVC laterality				
Ipsilateral (%)	15 (31.3)	NA	15 (68.2)	
Contralateral (%)	3 (6.3)	NA	3 (13.6)	
Bilateral (%)	4 (8.3)	NA	4 (18.2)	
Psychiatric history				
None (%)^†^	15 (31.3)	7 (26.9)	8 (36.4)	1.000
Schizophrenia	1	0	1	
Bipolar disorder	4	3	1	
Depressive disorder	11	6	5	
Anxiety disorder	12	7	4	
Obsessive–compulsive disorder	1	1	0	
Somatic symptom disorder	7	5	2	
Insomnia disorder	2	0	2	
Other	1	1	0	
Detail is unknown	3	2	1	
Comorbid oral psychosomatic symptom				
None (%)^†^	31 (64.6)	14 (53.8)	17 (77.3)	0.132
Burning mouth syndrome	6	3	3	
PIFP (atypical odontalgia)	8	8	0	
Phantom bite syndrome	1	1	0	
Others	2	0	2	
Symptom triggers				
None (%)^†^	20 (44.2)	12 (48.1)	8 (40.0)	0.588
Dental caries treatment	9	5	4	
Tooth extraction	6	3	3	
Prosthodontic treatment	7	4	3	
Periodontal treatment	2	1	1	
Denture adjustment	2	1	1	
Occlusal adjustment	1	0	1	
Implant placement	1	0	1	
Questionnaires (mean ± SD)				
SDS^§^	46.5 ± 9.9	47.2 ± 10.3	45.8 ± 9.6	0.645
PCS^§^	28.2 ± 15.2	31.2 ± 12.5	24.6 ± 17.4	0.132
SSS-8^§^	6.9 ± 4.2	7.3 ± 3.8	6.4 ± 4.6	0.464
SIUS^§^	30.7 ± 10.5	31.0 ± 10.3	30.2 ± 10.9	0.793
STarT-G^§^	2.2 ± 1.3	2.4 ± 1.1	1.9 ± 1.4	0.184

NVC of trigeminal nerves was observed in 22 (45.8%) patients having predominantly unilateral OC. There was no significant between-group difference in sex, age, illness duration, CGI severity, presence of psychiatric history, presence of comorbid oral psychosomatic symptoms, presence of symptom triggers and psychological questionnaires. NVC, neurovascular contact; PIFP, persistent idiopathic facial pain; CGI, Clinical Global Impression; SD, standard deviation; SDS, Zung Self-Rating Depression Scale; PCS, Pain Catastrophizing Scale; SSS-8, Somatic Symptom Scale 8; SIUS, Short Intolerance of Uncertainty Scale; STarT-G, subgrouping for targeted treatment generic; NA, not applicable. *p < 0.05.

† *Fisher's exact test*.

‡ *Mann–Whitney U-test*.

§ *Student's t-test*.

### Comparisons of the Questionnaire Scores

**Figure 3:**
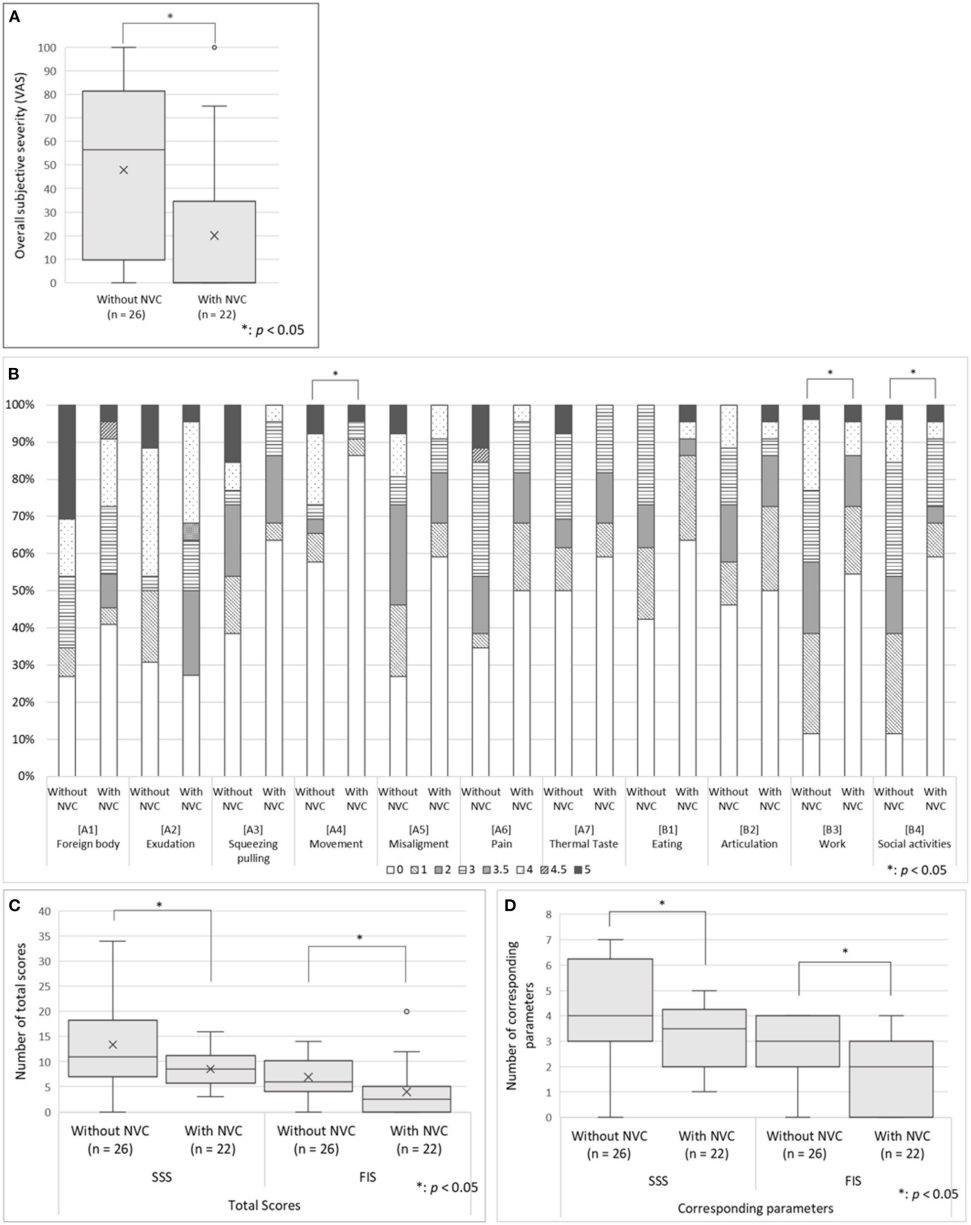
The oral DRS scores in the patients having predominantly unilateral OC with and without NVC. **(A)** The overall subjective severity of OC symptoms: The patients without NVC presented significantly higher VAS scores that indicate overall subjective severity of OC symptoms according to Student's *t*-test. **(B)** The Oral DRS scores: The patients without NVC showed significantly higher scores in the parameters of movement, work, and social activities than patients with NVC. Mann–Whitney U-test was used for each parameter. **(C)** The total scores of corresponding categories in the SSS and FIS: The patients without NVC showed significantly higher averages of the total scores and the number of corresponding symptoms in both the SSS and FIS than the patients with NVC based on Student's *t*-test. **(D)** Total number of corresponding categories in the SSS and FIS: The patients without NVC showed significantly higher number of the total number of corresponding symptoms in both the SSS and FIS than the patients with NVC according to Mann–Whitney U-test. For the box-and-whisker plot in panels **(A,C,D)** the line in the middle indicates the median; the top and bottom lines show the first and third quartiles. The cross marks show the mean values, and the whiskers extend to 1.5 times the height of the box or, if no case/row has a value in that range, to the minimum or maximum values. The points are outliers. VAS, Visual Analog Scale; Oral DRS, Oral Dysesthesia Rating Scale; OC, oral cenesthopathy; NVC, neurovascular contact; SSS, symptom severity scale; FIS, functional impairment scale.

There was no significant between-group difference in the scores of the SDS, PCS, SSS-8, SIUS, and STarT-G (Table 1); however, the VAS scores that indicate overall subjective severity of OC symptoms were significantly higher in the patients without NVC (47.8 ± 35.4 vs. 20.0 ± 34.3, *p* = 0.008; Figure 3A). Although there was no significant between-group difference in the pain [A6] parameter of the Oral DRS, the patients without NVC had significantly higher scores in the movement [A4] [0.0 (0.0–3.75) vs. 0.0 (0.0–0.0), *p* = 0.030], work [B3] [2.0 (1.0–3.0) vs. 0.0 (0.0–1.75), *p* = 0.004], and social activities [B4] [2.0 (1.0–3.0) vs. 0.0 (0.0–2.75), *p* = 0.010] parameters than the patients with NVC (Figure 3B). Moreover, compared with patients with NVC, patients without NVC showed significantly higher averages of the total SSS and FIS scores in the Oral DRS (13.4 ± 8.4 vs. 8.5 ± 3.7, *p* = 0.015; 6.9 ± 4.0 vs. 4.0 ± 5.0, *p* = 0.031; respectively; Figure 3C). Furthermore, compared with patients with NVC, patients without NVC had significantly higher numbers of corresponding symptoms in both the SSS and FIS [4.0 (3.0–6.0) vs. 3.5 (2.0–4.0), *p* = 0.041; 3.0 (2.0–4.0) vs. 2.0 (0.0–3.0), *p* = 0.007, respectively; Figure 3D].

## Discussion

This is the first study to investigate the relationship between the clinical characteristics of the patients having predominantly unilateral OC and the presence of trigeminal nerve NVC based on MRI findings. NVC of trigeminal nerves was observed in 45.8% of the patients; moreover, patients without NVC showed more complex symptoms and severe dysfunction in their daily lives.

Patients having OC present complaints of various uncomfortable or abnormal sensations in the oral cavity, without dental and medical evidence. Moreover, OC symptoms show various severities, generally intractable, and the treatment efficacy is limited (2). OC has often been regarded as DDST; however, only one case of schizophrenia was observed but no DDST in the present study. OC may tend to be diagnosed as somatic symptom disorder (SSD) rather than DDST, since SSD was diagnosed in seven patients.

Previous studies have suggested the involvement of the peripheral nervous system and CNS in the pathological process of abnormal oral sensations. Since trigeminal nerves transmit not only pain but also thermal, tactile, and pressure sensations, NVC of trigeminal nerves could also contribute to abnormal sensations in patients having OC. In the present study, 45.8% of the patients having predominantly unilateral OC showed NVC, which was a similar rate as that observed in our previous study on patients with unilateral PIFP (10). In patients having unilateral OC with NVC, the stimulus at the REZ is transmitted to the CNS *via* the thalamus, where it is simply recognized as pain or uncomfortable sensations. NVC in OC patients may not relate to induce pain sensations like trigeminal neuralgia but may induce indescribable subtle oral symptoms. Notably, in the comorbid oral pain parameter of the Oral DRS, there was no significant difference between the OC patients with and without NVC. Rather, more OC patients without NVC were corresponded in pain parameter and showed more severe pain compared to the patients with NVC. Moreover, the patients without NVC showed significantly more complicated oral symptoms with higher symptom severity and functional impairment, which decreased the quality of life. While NVC may affect to induce indescribable subtle OC symptoms, other more complex mechanisms may exist. Since there was no significant between-group difference in the comorbid psychiatric history and the psychometric questionnaire scores in the present study, OC symptoms may not be simply affected by the psychiatric or the psychological backgrounds. Previous studies using SPECT have suggested that right-dominant asymmetric CBF in the temporal region affects OC symptoms (7, 8). The sensory processing, as well as recognition performance in several cortical and subcortical brain regions, is extremely complex. Dysfunction in these regions may induce errors in normal sensory processing. Therefore, for patients having OC without NVC, there may be some mechanisms involving the CNS that make symptoms vary widely and become complex.

In addition, most patients with trigeminal neuralgia complain unilateral symptoms, but the laterality, whether left or right, is not significant (9). On the other hand, 268/348 OC patients showed bilateral OC symptoms who were excluded from the present study, and the left-dominant oral symptoms were found in 64.0% patients with predominantly unilateral OC. Although no significance was found in this study due to the small sample size, the left-dominant OC symptom and the ipsilateral NVC would be key points for the next investigations considering right-dominant asymmetric CBF in patients with OC (7, 8). The NVC laterality also should be investigated with a larger sample size.

Further studies on both the CNS and peripheral nervous system are required to investigate the highly complex pathophysiology of OC.

The present study has several limitations. First, the small sample size and biased participants would be confounders. The participants in the specialized department might be a bias, although it would also be a strength to support investigation for the primary oral psychosomatic disorder because all patients were referred from attending physicians, otolaryngologists, psychiatrists, and so on, besides the referral from dentists after the exclusion of systemic and organic etiology. Second, we did not assess CNS involvement. Other than SPECT, diffusion tensor imaging could be useful for investigating nerve fiber connectivity in both the peripheral nervous system and CNS (22).

In this study, NVC of trigeminal nerves was observed in about half of the patients having predominantly unilateral OC. Moreover, the OC patients without NVC presented more complex oral symptoms as well as significantly higher severity and dysfunction in their daily activities than patients with NVC. These results suggest that while NVC affects to induce indescribable unpleasant OC symptoms, the more complex mechanisms may exist in OC patients without NVC, which may yield widely varying and more unbearable oral symptoms.

## Data Availability Statement

The original contributions presented in the study are included in the article, further inquiries can be directed to the corresponding author.

## Ethics Statement

The studies involving human participants were reviewed and approved by the Ethical Committee of Tokyo Medical and Dental University. The patients provided their written informed consent to participate in this study.

## Author Contributions

KW participated in data acquisition and analysis and writing first draft preparation. MW verified the analysis and wrote, reviewed, and edited the manuscript. TS, TT, CH, ZL, CT, TY, and MT participated in collecting data. JS and TK contributed to data acquisition and analysis of imaging data. YU, AU, HM, YA, and AT developed the theory and supervised the research. AT designed the research and administered the project. All authors read and approved the final article.

## Funding

This research was funded by KAKENHI from the Japanese Society for the Promotion of Science (JSPS) Grant Number 19K10328 to AT and Grant Number 19K19211 to MW. The funder was not involved in the study design, collection, analysis, interpretation of data, the writing of this article or the decision to submit it for publication.

## Conflict of Interest

The authors declare that the research was conducted in the absence of any commercial or financial relationships that could be construed as a potential conflict of interest.

## Publisher's Note

All claims expressed in this article are solely those of the authors and do not necessarily represent those of their affiliated organizations, or those of the publisher, the editors and the reviewers. Any product that may be evaluated in this article, or claim that may be made by its manufacturer, is not guaranteed or endorsed by the publisher.

## Abbreviations

OC: oral cenesthopathy
BMS: burning mouth syndrome
DDST: delusional disorder somatic type
Oral DRS: Oral Dysesthesia Rating Scale
CNS: central nervous system
SPECT: single-photon emission computed tomography
CBF: cerebral blood flow
REZ: root entry zone
NVC: neurovascular contact
PIFP: persistent idiopathic facial pain
MRI: magnetic resonance imaging
MRA: MR angiography
3D-TOF: 3D time-of-flight
TR: repetition time
TE: echo time
FOV: field of view
3D-CISS: 3D constructive interference in steady-state
SSS: symptom severity scale
FIS: functional impairment scale
VAS: visual analog scale
SDS: Zung Self-Rating Depression Scale
SIUS: the Short Intolerance of Uncertainty Scale
SSS-8: Somatic Symptom Scale 8
PCS: Pain Catastrophizing Scale
STarT-G: subgrouping for targeted treatment generic
CGI: Clinical Global Impression
SD: standard deviation
IQR: interquartile range
SSD: somatic symptom disorder.
